# Treatment options for patients with hormone receptor-positive, HER2-negative advanced-stage breast cancer: maintaining cyclin-dependent kinase 4/6 inhibitors beyond progression

**DOI:** 10.3389/fonc.2023.1272602

**Published:** 2023-10-04

**Authors:** Malek Horani, Hikmat Abdel-Razeq

**Affiliations:** ^1^ Department of Internal Medicine, King Hussein Cancer Center, Amman, Jordan; ^2^ School of Medicine, the University of Jordan, Amman, Jordan

**Keywords:** CDK4/6 inhibitors, palbociclib, ribociclib, abemaciclib, metastatic breast cancer, disease progression, endocrine therapy

## Abstract

Breast cancer is the most commonly diagnosed cancer in women worldwide. Over the past decade, the treatment paradigm for patients with metastatic breast cancer (MBC) has taken an important shift towards better survival and improved quality of life (QOL), especially for those with hormone receptor (HR)-positive diseases which represent the majority of breast cancer subtypes. The introduction of cyclin-dependent kinase 4/6 (CDK4/6) inhibitors in the upfront therapy of such patients has resulted in dramatic improvement in progression-free survival (PFS) and overall survival (OS), too. However, almost all patients would, sooner or later, develop disease progression and necessitate transition to different lines of treatment that may include chemotherapy. The idea of maintaining CDK4/6 inhibitors beyond disease progression seems attractive, as this approach has the potential to improve outcome in this setting despite the fact that the true benefit, in terms of survival, might not carry the same weight as it initially does. Researchers have been investigating potential mechanisms of resistance and identify possible biological markers for response after disease progression. Much of the available data is retrospective; however, few randomized clinical trials were recently published and few more are ongoing, addressing this point. In this paper, we intend to review the available published studies investigating the potential role for keeping CDK4/6 inhibitors in play beyond disease progression.

## 1 Introduction

Breast cancer is the most prevalent cancer in women worldwide and one of the leading causes of death among women in the United States and worldwide (1–3). Patients with advanced breast cancer may present with *de novo* metastatic disease in a proportion of patients that varies in different health care systems, significantly more in low-income countries (4). Additionally, a sizable proportion of patients may progress to advanced stages following treatment of early or locally advanced diseases (5).

The majority of breast cancer patients belong to HR+/HER2− subtype (6), which carries a more favorable prognosis compared to the other subtypes (7). Over the years, chemotherapy and endocrine therapy (ET) had been the mainstay of treatment of advanced HR+/HER2− breast cancer. The addition of cyclin-dependent kinase 4/6 (CDK4/6) inhibitors to ET in the treatment of advanced-stage breast cancer has boosted responses and survival outcomes over the past few years, especially in the first-line setting (8). Ribociclib, palbociclib, and abemaciclib have all been approved, based on better disease control and survival benefits when combined with ET and have become the standard of care as first-line treatment for advanced HR+/HER2− breast cancer (9–11). CDK4/6 inhibitors have also produced significant improvements and better outcomes in second-line settings when combined with fulvestrant upon progression on aromatase inhibitor (AI) (12). In this manuscript, we review previous attempts and ongoing trials investigating the role of continuing the same or different CDK4/6 inhibitors, with ET, beyond disease progression.

## 2 Systemic therapies following progression on CDK4/6-inhibitors:

### 2.1 Analysis of real-world data

Patients with advanced HR+/HER2− breast cancer whose disease has progressed on frontline CDK4/6 inhibitors with ET have many options for treatment, but no standard of care exists for the next line of systemic therapy. Possible strategies include switching to different class of ET, switching to chemotherapy, as single agent or in combination, or utilizing novel targeted agents. Agents like alpelisib for patients with somatic PIK3CA mutations; elacestrant, a newly approved selective estrogen receptor degrader (SERD); everolimus; a mammalian target of rapamycin [mTOR] inhibitor; and poly (ADP-ribose) polymerase (PARP) inhibitors like talazoparib or olaparib for patients with germline BRCA1 or BRCA2 mutations are widely used (13–16). The optimal sequencing of the above options is not well-established; however, the choice of the next line of treatment depends on many factors including underlying comorbidities, menopausal status, potential adverse effects, molecular profile, presence of specific germline mutations, and the presence or absence of solid indications to start cytotoxic chemotherapy, in addition to patients’ preference.

The idea of CDK4/6 inhibitor continuation beyond progression was first studied in several small retrospective studies. In one study, analysis was done on 30 female patients with HR+/HER2-negative MBC treated at the Cleveland Clinic Foundation, who continued CDK4/6 inhibitors after initial progression. The primary endpoint was progression-free survival (PFS) beyond first documented disease progression. Initial ET-CDK4/6 inhibitor regimens received included palbociclib combined with letrozole (67%), fulvestrant (23%), or other ET. Only a minority of patients were on abemaciclib combinations. The median PFS for all patients while receiving CDK4/6 inhibitors and ET combination was 23.5 months (95% CI, 12.8–27.8), and median PFS beyond initial progression was 11.8 months (95% CI 5.34–13.13). Median OS since treatment initiation was around 45.4 months (17).

Two years later, another report was published with a similar concept. The analysis included 87 patients with metastatic HR+/HER2-negative patients who received palbociclib-containing regimens in the metastatic setting and were rechallenged with abemaciclib in combination with ET on progression (18). Palbociclib was combined with AI in the majority of patients (63%); the rest had it combined with fulvestrant. Approximately, a third (36.8%) of the patients switched to fulvestrant and abemaciclib after disease progression on AI and palbociclib. The same ET (AI or fulvestrant) was maintained with switching the CDK4/6 inhibitor to abemaciclib in around 25% of the patients. Only a minority of patients switched to abemaciclib monotherapy. Median PFS was similar for patients who received abemaciclib combined with an ET (5.1 months, 95% CI, 3.2–7.6) compared with patients who received abemaciclib as monotherapy (5.4 months, 95% CI, 1.9–NR). In order to further investigate the potential benefit of abemaciclib, another analysis was done on patients based on treatment with an ET to which they were not exposed, compared to rechallenging with ET with a previous exposure. There were no meaningful differences in both PFS (5.1 vs. 5.7 months) and OS (17.2 vs. 15.3 months). In terms of CDK4/6 inhibitor sequencing and its effect on outcome, median PFS was better in patients receiving sequential CDK4/6 inhibitors (8.4 months, 95% CI, 4.1–NR) compared to 3.9 months (95% CI, 2.9–5.7) in patients receiving non-sequential CDK4/6 inhibitor treatment (p = 0.0013) (18). However, one cannot make conclusions based on these statistics as patients on the non-sequential approach would have probably had a more aggressive disease. RB1 alterations and ERBB2 and CCNE1 amplification were detected by gene sequencing in few patients who developed rapid disease progression on CDK4/6 inhibitors; those mutations could be an early indicator for lack of efficacy and primary resistance the CDK4/6 inhibitor class (18).

A recently published analysis of real-world data was conducted at two centers in the United States to determine what systemic therapies were being used following progression on a CDK4/6 inhibitor and compare differences in outcome (19). This study was designed to investigate systemic therapies used in the second-line setting following disease progression on first-line ET-CDK4/6 inhibitor combinations. It also aimed to describe the real-world PFS (RW-PFS) and OS after initiation of second-line modalities. In the analysis, palbociclib was the CDK4/6 inhibitor used in the majority of patients in the first-line setting (88.2%) while the remaining received either ribociclib or abemaciclib. Aromatase inhibitors were the companion ET in around two-thirds of the patients, and fulvestrant with the other third. A total of 839 patients eventually received second-line systemic therapy and were included in the analysis. The most common second-line therapy was chemotherapy (29.7%), while ET monotherapy was used in 12.4% of the patients, most of which were treated with fulvestrant. The analysis also showed use of targeted agents, like everolimus, in 11.7%, while few others used PARP inhibitors or alpelisib (19). A CDK4/6 inhibitor was continued, alone or combination with ET as a second line, in 302 patients; most of them maintained the same CDK4/6 inhibitors used initially. For patients receiving a CDK4/6 inhibitor in the second-line treatment, the median OS was 35.7 months and the median RW-PFS was 8.25 months. For patients treated with chemotherapy, fulvestrant as single agent, or everolimus, the estimated median RW-PFS was worse: 3.71, 3.25, and 3.32 months, respectively. RW-PFS was significantly better with CDK4/6 inhibitor continuation when it was compared to chemotherapy (HR 0.48, 95% CI 0.43–0.53, p < 0.0001), as OS analysis showed benefit with CDK4/6 inhibitor continuation as well (HR 0.30, 95% CI, 0.26–0.35, p < 0.0001) (19).

More recently, another real-world data analysis was published from Japan, as investigators explored treatment modalities and their effect on subsequent therapy lines following disease progression on palbociclib-based combinations. Time to treatment failure (TTF) was the main endpoint (20). Three different approaches of CDK4/6 inhibitor sequencing were undertaken. First, both CDK4/6 inhibitor and ET were switched (i.e., palbociclib was replaced by abemaciclib and ET was switched to another agent). Second, only the ET was switched while palbociclib was maintained. Third, only the CDK4/6 inhibitor was switched (abemaciclib replaced palbociclib) while ET was maintained. The analysis included 1,170 patients treated with palbociclib combinations in the first-line setting and beyond. The combination of fulvestrant and abemaciclib was the most commonly used subsequent therapy. Median TTF of the first subsequent ET (as single agent) was 4.4 months (95% CI, 2.8–13.7) while patients on CDK4/6 inhibitor and ET combinations had a TTF of 10.9 months (95% CI, 6.5–15.6). Patients treated with ET and mTOR inhibitor combination had a TTF of 6.1 months (95% CI, 5.1–7.2). A subgroup analysis based on ET-therapy sensitivity showed that TTF for the ET-CDK4/6 inhibitor combinations was relatively long in both ET-sensitive and ET-resistant subgroups (20).

These observational data suggest that it is not uncommon for physicians to proceed with the same or different CDK4/6 inhibitor upon progression on their prior ET-CDK4/6 inhibitor combinations. **Table 1** summarizes the outcomes of the abovementioned studies.

**Table 1:** Summary of non-randomized trials.

Study (reference)	Number of patients	Initial CDK4/6 inhibitor regimen	Primary Endpoint	Arms	Outcome
Samuel Eziokwu A, et al. Retrospective Analysis (17)	30	Palbociclib-containing regimen	PFS*	CDK4/6 inhibitor + switch ET	11.8 months (95% CI, 5.34–13.13)
Wander SA, et al. Retrospective Analysis (18)	87	Palbociclib–AI Palbociclib–fulvestrant	PFS*	Abemaciclib monotherapy	5.4 months (95% CI, 1.9–NR)
				Abemaciclib + ET	5.1 months (95% CI, 3.2–7.6)
				Sequential CDK4/6 inhibitor	8.4 months (95% CI, 4.1–NR)
				Non-sequential CDK4/6 inhibitor	3.9 months^^^
Martin JM et al. Analysis of Real-World data-US (19)	839	Palbociclib (88%), ribociclib, or abemaciclib (12%) AI (2/3) Fulvestrant (1/3)	RW-PFS*	CKD4/6 inhibitor (+/− ET)^#^	8.25 months^^^
				Chemotherapy	3.71 months^^^
				Fulvestrant monotherapy	3.25 months^^^
				Everolimus	3.32 months^^^
Masataka Sawaki, et al Analysis of Real-World data -Japan (20)	1,170	Palbociclib-based regimens	TTF	Endocrine monotherapy	4.4 months (95% CI, 2.8–13.7)
				CKD4/6 inhibitor + ET	10.9 months (95% CI, 6.5–15.6)
				ET + mTOR inhibitor	6.1 months (95% CI, 5.1–7.2)

PFS, progression-free survival; AI, aromatase inhibitors; ET, endocrine therapy; RW, real world; TTF, time to treatment failure.

*Beyond initial progression.

^#^Versus chemotherapy: HR 0.48, 95% CI 0.43–0.53.

^95% CI not reported in the original study.

## 3 Systemic therapies following progression on first-line CDK4/6 inhibitors:

### 3.1 Randomized trials

Three randomized clinical trials trying to answer the same question were recently published. The first was the MAINTAIN trial which is a randomized phase II trial studying the efficacy of maintaining palbociclib with or without ET in patients whose disease had progressed on ET+CDK4/6 inhibitor (21, 22). A total of 119 patients with metastatic HR+/HER2-negative breast cancer (patients could have received up to one line of chemotherapy) were included in the study and were randomized into two arms: the first received (switch) ET combined with ribociclib, and the other arm (switch) ET combined with placebo (60 and 59 patients, respectively); the initial CDK4/6 inhibitor used in the prior line was palbociclib in the majority of patients. Switch ET meant that patients receive fulvestrant as ET in the case of disease progression on a prior AI or receive AI (exemestane) in the case of disease progression on fulvestrant. PFS was the primary endpoint of the study; secondary endpoints included overall response rate (ORR) and OS, among others (22). At data cutoff with a median follow-up of 18 months, PFS was improved in the ribociclib arm when compared to placebo, 5.29 months vs. 2.76 months, respectively, with a hazard ratio of 0.57 and a 95% CI of 0.39–0.95 and a significant p-value of 0.006. Median PFS at 12 months was also improved, 24.6% for the combination arm versus 7.4% for the placebo arm (22).

#### 3.1.1 The addition of immunotherapy

The addition of immunotherapy to the combination of ET+CDK4/6 inhibitors was studied in the PACE trial, which was a multicenter randomized open-label phase III trial conducted prospectively to study the efficacy of palbociclib continuation combined with fulvestrant beyond disease progression on prior AI+CDK4/6 inhibitors, compared to fulvestrant monotherapy, and to study the role of adding immunotherapy (avelumab) to the palbociclib/fulvestrant combination (23). There were a total of 220 patients with metastatic HR+/HER2-negative breast cancer with prior progression on AI and any CDK4/6 inhibitors. Similar to the MAINTAIN trial, patients could have been treated with only one line of chemotherapy in the metastatic setting. Palbociclib was the initial CDK4/6 inhibitor in the vast majority of patients. PFS (palbociclib/fulvestrant vs. fulvestrant monotherapy) was the primary endpoint. PFS for the triplet combination (versus fulvestrant monotherapy) was a secondary endpoint, in addition to objective response rate across all arms (24). In regard to the primary endpoint after a median follow-up of 2 years, the palbociclib combination failed to show benefit as the PFS for the palbociclib/fulvestrant arm was 4.6 months and 4.8 months for the fulvestrant monotherapy arm (HR = 1.11 and a two-sided p-value of 0.62). As for the secondary endpoints, median PFS was numerically better in the triplet arm (8.1 months) but was not statistically significant (hazard ratio of 0.75 vs fulvestrant monotherapy, and a two-sided p-value of 0.23). The overall response rates were 7.3% for the fulvestrant monotherapy arm, 9% for the doublet (fulvestrant and palbociclib) combinations, and 13% for the triplet combinations. The clinical benefit rates were more or less similar between all arms. Adverse effects were consistent with the safety profile accustomed to each agent (24).

Finally, the PALMIRA trial, which was an international, multicenter, randomized, open-label, phase II trial was conducted, aiming to evaluate the efficacy of continuation of palbociclib combined with second-line ET in patients with HR+/HER2− advanced breast cancer after disease progression on palbociclib-based first-line combination with ET (25). The analysis included 198 patients who were eligible if they had evidence of clinical benefit to ET+CDK4/6 inhibitors in the first-line setting (i.e., no primary endocrine resistance). Patients were randomly assigned to receive either palbociclib combined with switch ET (fulvestrant or letrozole) or second-line switch ET monotherapy. PFS was the primary endpoint of the trial, secondary endpoints included clinical benefit rate and overall response, among others (26). At data cutoff and after a median follow-up of 8.7 months, median PFS for the two arms were similar, 4.2 months and 3.6 months in the palbociclib/ET and ET monotherapy arms, respectively. Overall response and clinical benefit rates were also similar in the two arms. In terms of safety, the combination arm had more grade 3/4 toxicity (45.2% vs. 8.3%) (26). **Table 2** shows a summary of all three trials.

**Table 2:** Randomized studies comparing CDK4/6 extension beyond progression versus other treatment options.

Study (Reference)	Study design (Number of patients)	Initial CDK4/6 inhibitor regimen	Median follow-up (months)	Arms	PFS^*^ (Months)	HR, p-value, 95% CI
**MAINTAIN** (21, 22)** **	Randomized phase II trial (n = 119)	Palbociclib + AI/fulvestrant	18	Switch ET + switch to ribociclib	5.29 (95% CI 3.02–8.12)	HR 0.57, (95% CI 0.39–0.95) p = 0.006
				Switch ET + placebo	2.76 (95% CI 2.66–3.25)	
**PACE** (23, 24)	Randomized open-label, phase III trial (n = 220)	Any CDK4/6 inhibitor^#^ + AI	24	Fulvestrant + palbociclib	4.8^^^	HR = 1.11 (90% CI 0.79–1.55) Two-sided p = 0.62
				Fulvestrant monotherapy	4.6^^^	
				Fulvestrant + palbociclib + avelumab	8.1^^^	HR = 0.75 (vs fulvestrant monotherapy) (90% CI 0.50–1.12) Two-sided p = 0.23
**PALMIRA** (25, 26)	Randomized, open-label, phase II trial (n = 198)	Palbociclib + ET	8.7	Switch ET + palbociclib	4.2 (95% CI 3.5–5.8)	HR 0.8 (95% CI 0.6–1.1) p = 0.206
				Switch ET monotherapy	3.6 (95% CI 2.7–4.2)	

PFS, progression-free survival; HR, hazard ratio; CI, confidence interval; ET, endocrine therapy; AI, aromatase inhibitors.

*Beyond initial progression.

^#^Mostly palbociclib.

^95% CI not reported in the original study.

## 4 Discussion

**Figure 1:**
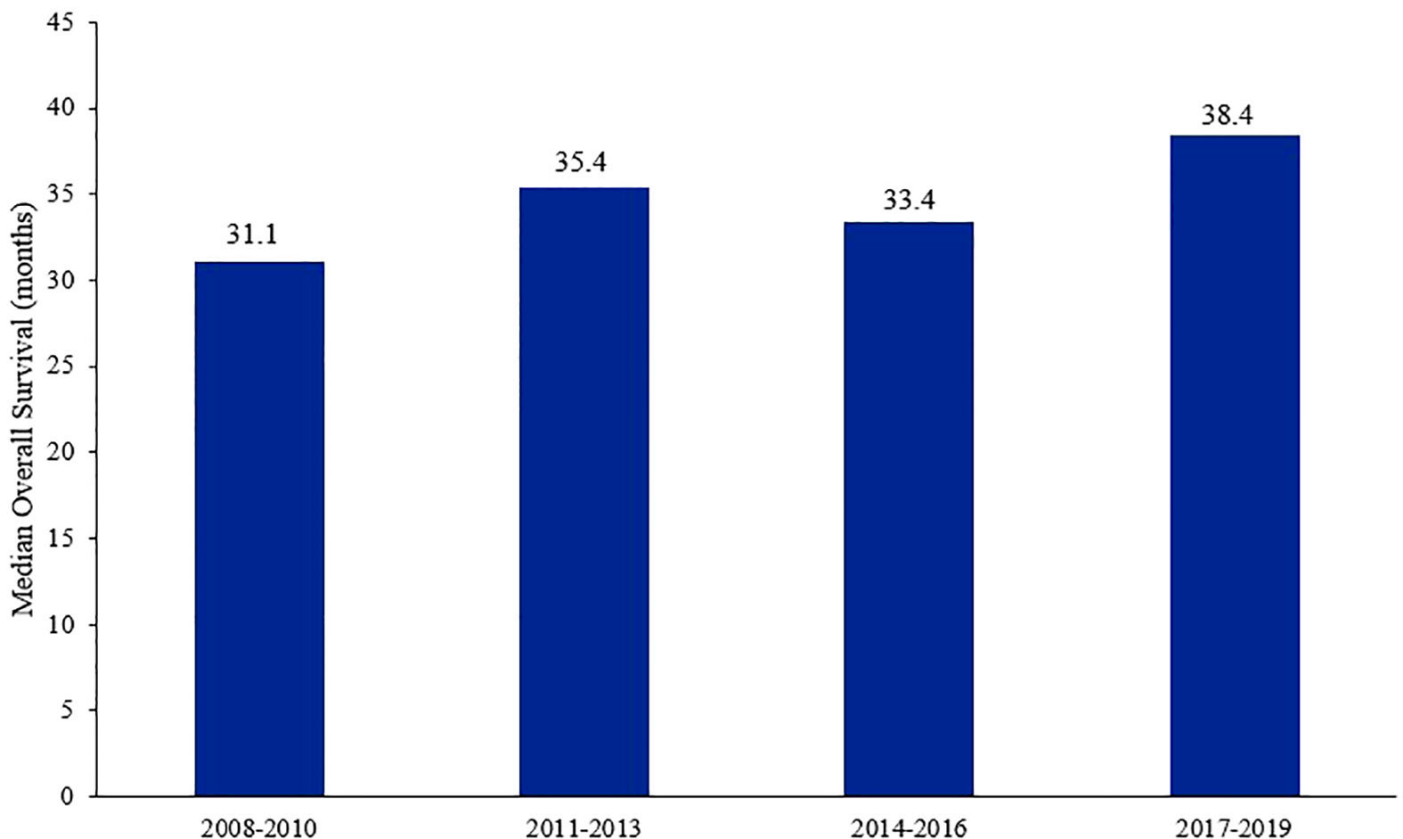
Median overall survival of patients with HR+/HER2− metastatic breast cancer over time.

Though the breast cancer-related mortality has decreased over the past few years (27), it remains one of the leading causes of death among women worldwide (3, 28). Treatment of breast cancer in the metastatic setting have come a long way in improving survival outcomes, especially in patients with HR+/HER2− tumors (**Figure 1**) (27). The addition of CDK4/6 inhibitors in the frontline setting, and even in subsequent lines after progression on ET, had impeccable results and have become the cornerstone in the treatment of such patients (29). Those drugs are generally well-tolerated (30); neutropenia, leukopenia, thrombocytopenia, anemia, fatigue, diarrhea, and transaminitis are the most frequent adverse effects encountered (31).

All CDK4/6 inhibitors have shown significant improvement in PFS, and some (ribociclib and abemaciclib) have also improved OS when combined with ET in both first- and second-line settings [9, 31]. The notion of maintaining CDK4/6 inhibitors after disease progression is intriguing, and that led many researchers at leading institutions around the world to report patients’ real-world outcomes, by switching the ET used and either maintaining the same CDK4/6 inhibitor or switching it to another. Most of the retrospective data discussed above were encouraging, suggesting that some patients may gain some benefit in maintaining CDK4/6 inhibitors upon progression on prior CDK4/6 inhibitor treatment. However, these data analyses were weak, as for their observational nature, inclusion of heavily pretreated patients, heterogeneous population, and in some, a small number of patients included. In addition, many of the clinical characteristics of treatment arms were lacking in some of these studies.

The MAINTAIN and PACE are two randomized clinical trials that investigated this approach, but the outcome was not the same leaving physicians with loose ends. In the MAINTAIN trial, both the CDK4/6 inhibitor and ET were switched upon progression and ribociclib was used after progression on palbociclib. Ribociclib combined with ET led to a statistically significant improvement in PFS. In an exploratory analysis, based on tumor biomarkers, the efficacy was better in patients who had no ESR1 mutation (ESR1 wild type); median PFS for the ESR1-WT treated with ribociclib was 8.3 months, compared to 2.7 months for those on placebo. Patients in both groups, with mutant ESR1, had similar PFS (32). This was a bit undermined by the small number in those subgroups; however, this would prove an eye opener for some of the following trials and future approaches in dealing with sequencing CDK4/6 inhibitors, and searching for other predictive biomarkers.

In the PACE trial, a different approach was undertaken as only ET was switched and the CDK4/6 inhibitor palbociclib was maintained in the majority of patients; in addition, a third arm was included with the addition of avelumab; a PD-L1 inhibitor. Maintaining palbociclib upon progression failed to prove beneficial in this trial, and the addition of immunotherapy (avelumab) showed PFS benefit but was not statistically significant; this might trigger more investigation in the near future.

Tumor biomarkers seemed to play an integral role in predicting response. Having certain mutations might carry a potential for more favorable response, as suggested by a subgroup analysis revealing that patients with PIK3CA and ESR1 mutations detected by liquid biopsy when analyzing circulating tumor DNA (ctDNA) had more favorable responses (33), making the argument to keep looking for predictive biomarkers even more powerful.

The PALMIRA trial, which is considered by many as the tiebreaker between the two previous trials, had also failed to demonstrate PFS benefit with palbociclib continuation. Further studies are ongoing to investigate the potential benefits of this approach. For now, the best course of action may will be sticking to other treatment modalities with proven better efficacy compared to ET monotherapy, including antibody–drug conjugates, targeted agents, or even chemotherapy.

It is worth-mentioning that none of the above trials experimented abemaciclib in the setting of progression beyond ribociclib or palbociclib. It seems that abemaciclib is different in terms of biological and potentially pharmacological characteristics than ribociclib and palbociclib (34), and this might justify switching to abemaciclib upon disease progression on a CDK4/6 inhibitor, which might have a potential role in overcoming resistance acquired to the previous CDK4/6 inhibitor. This approach is being evaluated in the ongoing post-MONARCH phase III trial (35).

Patients with early progression on CDK4/6 inhibitors (defined as disease progression in <6 months) might not be the best candidates for CDK4/6 inhibitors in subsequent lines as many of these patients would have some sort of primary resistance to this family of drugs (36), and potentially a more aggressive nature to the disease. In an attempt to investigate the possible pathways of resistance to CDK4/6 inhibitors, a phase III open-label multicenter trial (PADA-1 trial) was conducted in France investigating the possible implication of the ESR1 mutation on acquiring resistance to treatment in HR+/HER2− breast cancer (first randomized trial to do so). Patients with HR+/HER2− metastatic breast cancer were monitored for changes in ESR1 mutation in the ctDNA in blood while on palbociclib + AI combination therapy in the first-line setting (37). Randomization was based on detected ESR1 mutation status, as those patients with newly detected mutation or increasing mutation burden in the ctDNA with no evidence of disease progression were randomized to either continue with the same treatment or to switch to different ET-CDK4/6 inhibitor combination: fulvestrant with palbociclib. PFS was the primary endpoint in this trial. Out of the 1,000 patients initially recruited, 279 patients developed a rising ESR1 mutation. A total of 172 patients were randomized into two arms: 88 patients switching to the palbociclib + fulvestrant combination and 84 patients who were maintained on the same initial combination (palbociclib + AI). PFS estimated from random assignment in the intention-to-treat analysis was improved in the palbociclib + fulvestrant compared to the palbociclib + AI group (11·9 months vs. 5·7 months, respectively, with a hazard ration of 0·61, and a significant p-value 0·0040) (37).

The end result of the PADA-1 trial supports the approach that early therapeutic targeting of rising blood ESR1-mutation burden could carry significant clinical implications and has the potential benefit to predict primary resistance and possibly shorter survival. Around one-third of patients treated with the AI+CDK4/6 inhibitor combination will develop an ESR1 mutation at some point and subsequently develop resistance; however, there seems a good chance those patients would retain sensitivity to CDK4/6 inhibitors if the ET companion was changed (38). A recent phase II trial showed promising outcomes in patients with advanced HR+/HER2− breast cancer and acquired ESR mutation progressing on prior ET. In this small cohort trial, patients received treatment with a combination of abemaciclib and lasofoxifene (a non-selective estrogen receptor modulator). Most of the patients had disease progression on prior CDK4/6 inhibitor treatment; the median PFS was 13.9 months (95% CI, 8.0–NE), and the clinical benefit rate was 62.1% (39). An ongoing active phase III randomized trial (ELAINE-3) will evaluate the efficacy and safety of this combination against fulvestrant + abemaciclib in ESR1-mutated breast cancer (40).

It will be interesting to see more trials after PADA-1 with a similar design in the near future. To touch on that, an analysis update was recently published from the PACE trial in the most recent American Society of Clinical Oncology (ASCO) annual meeting (2023) (41), with monitoring the burden of circulating tumor cells (CTCs) in the blood, which was done at baseline, at time of first disease assessment, and finally at time of disease progression. Patients were classified into two categories according to the level of circulating tumor cells: indolent (<5 CTCs/7.5 ml) and aggressive (≥5 CTCs/7.5 ml). Baseline tumor cell readings were prognostic, as median PFS was 5.7 months for the indolent group and 3.5 months for the aggressive group. When the median PFS was estimated according to treatment groups, patients treated with fulvestrant monotherapy had PFS of 1.9 months for the “aggressive” group, compared to 8.5 months for the “indolent” ones, while the PFS for patients managed with fulvestrant/palbociclib combination was 4.6 months for the “aggressive” vs. 5.3 months for the indolent. Similarly, median PFS for patients managed with fulvestrant/palbociclib/avelumab triplet was 5.4 months in the “aggressive” vs. 8.3 months in the “indolent” (41). Further investigation of this model in the future or other similarly designed models might predict clinical benefit for either CDK4/6 inhibitor continuation or adding immunotherapy to the equation.

Secondary or acquired resistance to CDK4/6 inhibitors could result from various mutations including a mutation in RB1 leading to activation of other cell-cycle factors, such as E2F and the cyclin E-CDK2 axis. BioPER was a phase II trial exploring potential biomarkers (mainly Rb protein expression) for efficacy of continuing palbociclib beyond disease progression on prior palbociclib–ET combinations. A total of 32 patients were included in the final analysis with median follow-up around 18 months; the clinical benefit rate of maintaining palbociclib combined with physicians’ choice of endocrine therapy after disease progression on prior palbociclib-based combination, a primary endpoint, was 34.4% (95% CI, 18.6–53.2). PFS at 6 months was 31.2% (95% CI, 18.7–52.2). The percentage of patients with lost Rb protein expression (<1%) in tumor cells at baseline after disease progression was 13%, which was a biological coprimary endpoint. Treatment in those patients failed to achieve clinical benefit; this finding suggests that switching to another class of drugs might carry better chances for response (42, 43). An exploratory analysis showed significantly worse outcomes in patients with any of the following biomarkers detected: ESR mutation, low Rb protein expression, and high cyclin E1 expression. Detection of CTCs from liquid biopsies was done at different intervals during treatment; interestingly, undetected circulating tumor DNA at day 15 of cycle 1 was associated with significantly longer PFS.

Lastly, a better understanding of patterns of resistance driving loss of response to CDK4/6 inhibitor and/or ET will be essential to guiding more rational approaches and evidence-based selection of subsequent lines of treatment and improving outcomes for such patients. In addition, testing newer endocrine therapy agents that may possess different biochemical activity and potentially overcoming resistance to older-generation agents might help provide new options for treatment in patients with ET-resistant HR+/HER2− breast cancer, as an example; a phase III (EMBER 3) trial will evaluate the efficacy of a novel SERD “Imlunestrant” with or without abemaciclib, compared to investigator choice of ET in patients with disease progression beyond AI-CDK4/6 inhibitor combinations (44).

## 5 Conclusions

CDK4/6 inhibitors have changed the natural history of HR+/HER2− metastatic breast cancer. However, all patients will unfortunately progress and a new line of therapy should be introduced. Many drugs, as single agent or in combination, can be used in this setting. Our review showed that most of recently published clinical trials have failed to show meaningful improvement in outcome when CDK4/6 inhibitors continued following disease progression. However, the utilization of liquid biopsy to detect CTCs and ctDNA, and testing for certain biomarkers, may improve our ability to better select anticancer therapy following disease progression on CDK4/6 inhibitors.

## Author contributions

MH: Data curation, Writing – original draft, Writing – review & editing, Investigation. HA-R: Data curation, Writing – original draft, Writing – review & editing, Conceptualization, Methodology, Project administration, Supervision.
